# Automatic positive airway pressure for obstructive sleep apnea in heart failure with reduced ejection fraction

**DOI:** 10.1007/s00392-020-01701-1

**Published:** 2020-07-10

**Authors:** Henrik Fox, Thomas Bitter, Odile Sauzet, Volker Rudolph, Olaf Oldenburg

**Affiliations:** 1 Clinic for Thoracic and Cardiovascular Surgery, Herz- und Diabeteszentrum NRW, Ruhr-Universität Bochum, Georgstr. 11, 32545 Bad Oeynhausen, Germany; 2 Heart Failure Department, Herz- und Diabeteszentrum NRW, Ruhr-Universität Bochum, Bad Oeynhausen, Germany; 3 Clinic for General and Interventional Cardiology/Angiology, Herz- und Diabeteszentrum NRW, Ruhr-Universität Bochum, Bad Oeynhausen, Germany; 4 Epidemiology and International Public Health, Bielefeld School of Public Health and Statistical Consulting Centre, Bielefeld University, Bielefeld, Germany; 5 Department of Cardiology, Clemenshospital, Ludgerus-Kliniken, Münster, Germany

**Keywords:** Sleep-disordered breathing, Obstructive sleep apnea, Heart failure with reduced ejection fraction, Positive airway pressure

## Abstract

**Background:**

Moderate-to-severe obstructive sleep apnea (OSA) is highly prevalent in heart failure patients with reduced left ventricular ejection fraction (HFrEF), and is associated with worsening cardiac function and increased mortality.

**Objectives:**

The automatic positive airway pressure (APAP) trial tested the impact of APAP treatment on changes for the pre-specified endpoints: changes in peak oxygen uptake (peak VO_2_), percent-predicted peak VO_2_ and oxygen uptake at anaerobic threshold (VO_2_-AT).

**Methods:**

This randomized, controlled pilot study included patients with chronic, stable HFrEF who had moderate-to-severe OSA. Patients were randomized 1:1 to either APAP (AutoSet™, ResMed) or nasal strips (control) for 6 months.

**Results:**

76 patients have been randomized and 58 had complete data for final analysis. There was a statistically significant change in the APAP intervention arm for the primary endpoint percent-predicted peak VO_2_ in comparison to control (67 ± 17 to 73 ± 19%; *p* = 0.01). Additional primary endpoints peak VO_2_ and VO_2_-AT showed a trend in increase in the APAP group. Moreover, there were significant improvements within the APAP group for hypoxemia, left ventricular function and quality of life from baseline to 6 months, but not within the control group (*p* = 0.001 and *p* = 0.037, respectively).

**Conclusion:**

APAP intervention was shown to significantly improve outcome compared to control group, represented in percent-predicted peak VO_2_, an established surrogate marker for cardiovascular prognosis in HFrEF. APAP has additional beneficial effects on hypoxemia, cardiac function and quality of life.

## Introduction

Sleep-disordered breathing (SDB), obstructive sleep apnea (OSA) in particular, is highly prevalent in patients with heart failure (HF) [1, 2]. Epidemiological studies suggest that OSA is an independent risk factor for HF development and has a negative effect on prognosis in patients with HF [3–5]. OSA is characterized by repeated partial or complete collapse of the upper airway during sleep [6], accompanied by complete absence of airflow and paradoxical respiration with opposing respiratory movements of the thorax and abdomen [7]. Obstructive respiratory events result in negative intrathoracic pressure swings, which influence venous return and preload for the right ventricle, and trigger cardiac arrhythmias [8]. Another mechanism by which OSA might contribute to the pathophysiology of HF is by increasing sympathetic activation [9]. Endothelial dysfunction, inflammation, hypercoagulability and apoptosis have also been associated with OSA [10] and may contribute to the development or worsening of HF. Furthermore, nocturnal hypoxemia was a robust and independent predictor of all-cause mortality in patients with stable HF with reduced ejection fraction (HFrEF) in a cohort study [11].

Continuous positive airway pressure (CPAP) maintains upper airway patency by applying positive pressure during sleep [7, 12] and is the gold standard treatment for moderate-to-severe OSA in symptomatic patients with or without HF [7, 13]. Recently, CPAP has been shown to improve the clinical course of cardiac recompensation and to have a beneficial impact on pulmonary hypertension in patients with acute HF and OSA [14].

Automatically titrating CPAP (APAP) devices include an algorithm that adjusts delivered pressures to a patient’s individual demands. Patients with HF are expected to benefit from application of lower positive airway pressures delivered by APAP in terms of cardiac filling pressures and, especially, right ventricular function [15], making APAP an attractive option for the treatment of OSA in patients with HF. However, the randomized controlled SAVE (Sleep Apnea Cardiovascular Endpoints) trial of 2717 OSA patients with coronary or cerebrovascular disease did not show any significant beneficial effects of APAP on the rate of cardiovascular events and mortality over a mean follow-up of 3.7 years [16]. Hereby, device use during the trial was low, averaging only 3.3 h per night. Current literature suggests that positive airway pressure therapy for OSA needs to be used for a minimum of 4 h per night to achieve measurable benefits [7], including reductions in blood pressure [17] and prevention of recurrent atrial fibrillation [18].

Cardiopulmonary exercise capacity (peak VO_2_) is a well-known predictor of mortality in HFrEF and remains one of the major parameters defining qualification for heart transplantation [19]. Peak VO_2_, reflecting maximal oxygen consumption, is closely related to cardiac output, representing the best validated non-invasive parameter to classify HFrEF stage and severity [19]. However, there is a current lack of data on the impact of APAP therapy on exercise capacity in patients with HFrEF.

This randomized controlled pilot study investigated the impact of APAP therapy on cardiopulmonary exercise capacity, echocardiography measures of cardiac function, quality of life, and nocturnal OSA parameters in patients with HFrEF.

## Methods

### Study design

**Fig. 1:**
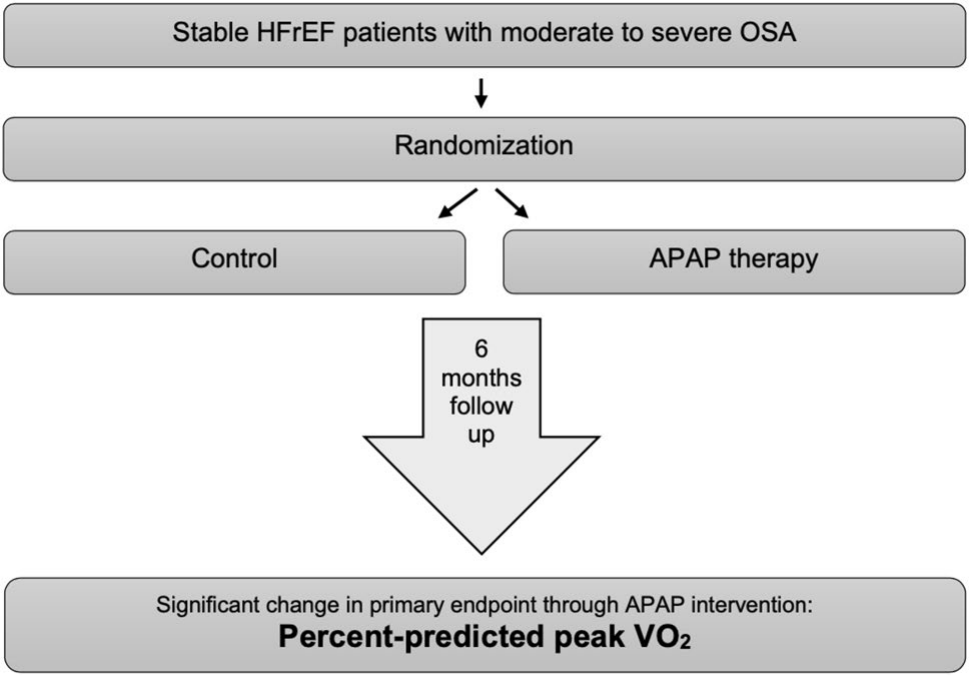
Study design and key finding. Intervention through APAP therapy for 6 months significantly improved primary endpoint percent-predicted peak VO_2_ in HFrEF patients with confirmed moderate-to-severe obstructive sleep apnea. *APAP* automatic positive airway pressure, *HFrEF* heart failure with reduced ejection fraction, *OSA* obstructive sleep apnea, *Peak VO*_*2*_ peak oxygen consumption

The Bad Oeynhausen APAP study is an investigator-initiated, prospective, randomized, controlled, parallel-group, open-label, blinded outcome, single-center interventional clinical trial comparing APAP (active treatment) and nasal strips (control group) for the treatment of OSA in patients with chronic, stable HFrEF receiving optimal medical therapy (Fig. 1) [13]. The study protocol was approved by the institutional ethics committee at Ruhr-University (Reg. No. 13/2008, approval date: 10th November 2008) and the trial is registered at Deutsches Register Klinischer Studien (German Clinical Trials Register; DRKS00000446). All patients were provided with information about the aim and nature of the trial by a cardiologist with expertise in sleep medicine. All procedures performed in this study involving human participants were in accordance with the ethical standards of the institutional research committee and with the 1964 Helsinki declaration and its later amendments or comparable ethical standards. After provision of written informed consent, patients were randomized in a 1:1 ratio by an independent coordinator using sealed envelopes without any prior stratification.

### Study patients

Screening for SDB in all HF patients has been routine clinical practice at our institution since 2003 [7]. Over the period of the study (2009 to 2016), patients with stable HFrEF in New York Heart Association (NYHA) functional class ≥ II and a left ventricular ejection fraction (LVEF) of ≤ 45%) were screened for the presence of OSA using unattended in-hospital overnight polygraphy; those who showed moderate-to-severe OSA in polygraphy recordings were invited to undergo full in-hospital polysomnography (PSG). Full details of study inclusion and exclusion criteria have been reported previously [13].

### Interventions

Patients randomized to APAP therapy were provided with an APAP device (AutoSet™ S8, S8II, S9 or AirCurve™; ResMed, Sydney, Australia). APAP treatment was started on the night immediately after PSG diagnosis and randomization, using full PSG and video surveillance for therapy titration. The interface used (nasal mask, full-face mask, or nasal pillows mask) was chosen based on patient/physician preference and the clinical indication to ensure optimal therapy application and adherence. Patients in the control group were discharged after overnight PSG and provided with nasal strips (Breathe Right^®^; GSK, United Kingdom) of various sizes. These are a proven placebo therapy in this context, and might reduce nasal resistance but have no effect on the degree of OSA or snoring in clinical trials [20].

### Hypothesis and predefined endpoints

These have been described in detail previously [13]. The primary endpoint is changes in peak VO_2_, percent-predicted peak VO_2_, and VO_2_ at anerobic threshold (VO_2_-AT) from baseline to 6 months change in cardiopulmonary exercise (CPX). The following secondary endpoints have also been assessed: change in 6-min walk distance (6MWD) from baseline to 3 and 6 months; changes in the Epworth sleepiness scale (ESS) and Minnesota living with heart failure (MLHF) questionnaire scores from baseline to 3 weeks, 3 months, and 6 months; echocardiographic measures of atrial and ventricular dimensions as well as right and left ventricular function after 3 and 6 months; change in the apnea–hypopnea index (AHI) and other SDB metrics; APAP therapy metrics including efficacy of APAP based on device-derived AHI and apnea index (AI) at 3 weeks, 3 months, and 6 months.

### Assessments and follow-up

Study visits took place at baseline, after 3 weeks as well as at 3 and 6 months of follow-up; baseline and 6-month visits were performed at hospital, while the other visits were outpatient appointments. The APAP device was interrogated and occurrence of serious adverse events were determined at all post-baseline visits. Cardiopulmonary exercise testing was undertaken at baseline, 3 months and 6 months. The same study visits also included the following: patient history, physical examination, NYHA class, echocardiography (conventional and speckle tracking analysis), 6MWD, blood sampling, and blood gas analysis. Blood pressure, heart rate and rhythm, MLHF questionnaire and ESS were evaluated at each study visit. Full details can be obtained in the study design paper [13].

### Sample size

Based on an expected mean peak VO_2_ of 17.0 ± 5.0 mL/kg/min, a clinically relevant between-group difference with peak VO_2_ of 10%, study power of 80% and an alpha value of 5%, the estimated sample size was calculated to be 70 patients [13].

### Statistical analysis

Statistical analysis was independently performed at Bielefeld Center for statistics. Continuous variables are reported as mean values with standard deviation, and categorical data as absolute and relative frequencies. The primary endpoint was evaluated in the per-protocol population (i.e. patients receiving treatment as intended throughout the study period). Change in the primary endpoints (changes in peak oxygen uptake (peak VO_2_), percent-predicted peak VO_2_ and oxygen uptake at individual anaerobic threshold (VO_2_-AT) during a standardized CPX test) from baseline to 6 months was determined using linear regression models with 6-month values as the dependent variable and therapy group with baseline values as the independent variable. Changes were analyzed within groups (from baseline to 6-month follow-up) and between groups at the 6-month follow-up. Other comparisons were performed on an explanatory basis, using paired or unpaired *t* test, Chi squared test, and non-parametric tests, as appropriate.

## Results

### Study population

**Fig. 2:**
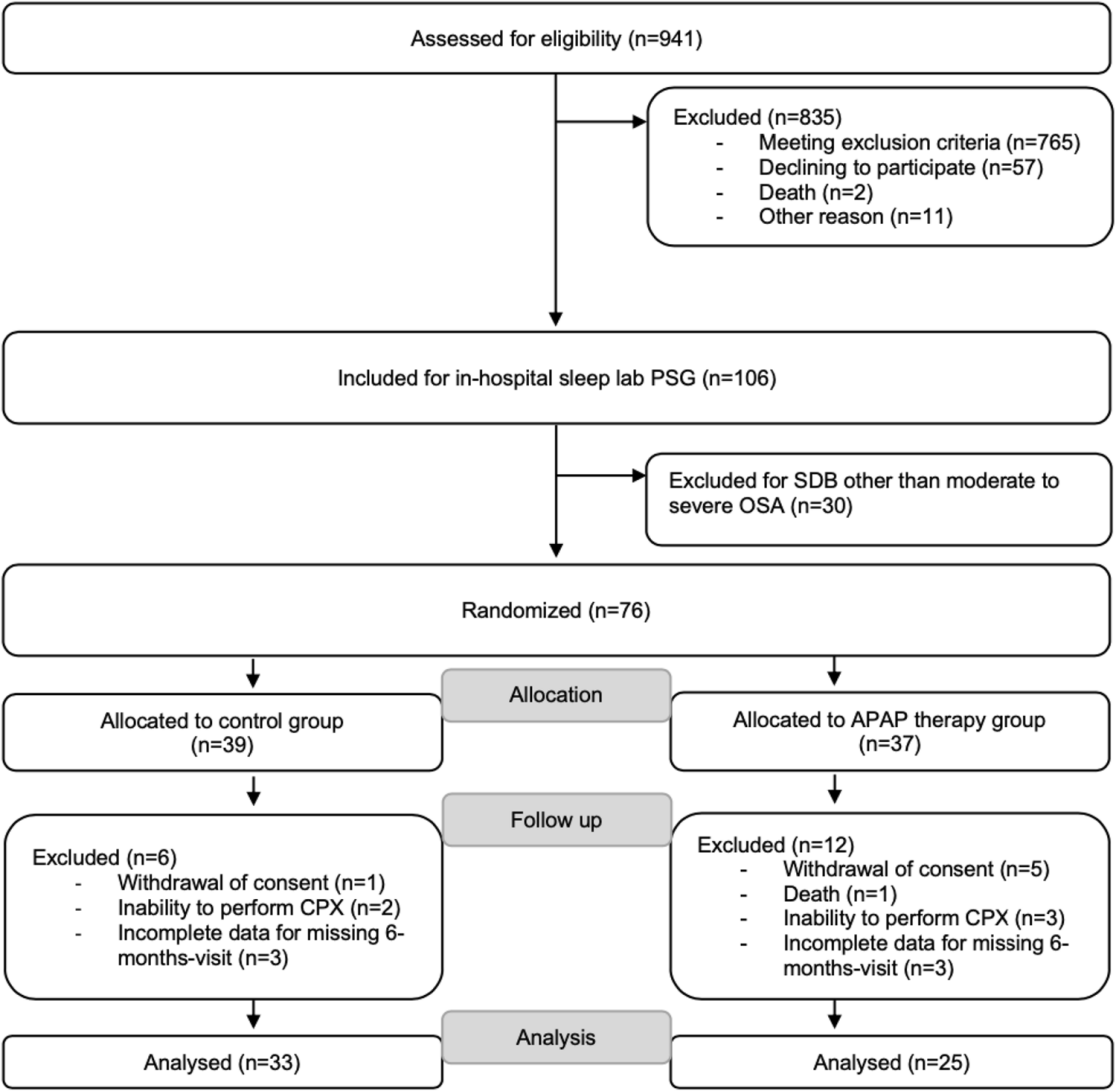
Patient flow. *APAP* automatic positive airway pressure, *CPX* cardiopulmonary exercise testing, *HFrEF* heart failure with reduced ejection fraction, *OSA* obstructive sleep apnea, *PSG* polysomnography, *SDB* sleep-disordered breathing. Exclusion criteria were: cardiac resynchronization therapy within the last 12 weeks, significant chronic obstructive pulmonary disease (forced expiratory volume in 1 s/vital capacity < 70%), respiratory insufficiency requiring home oxygen therapy, hypercapnia (pCO_2_ > 45 mmHg), current treatment with any kind of positive airway pressure therapy, relevant treatment-emergent central sleep apnea (complex sleep apnea), restless legs syndrome (untreated or non-treatable), any cardiac surgery and/or percutaneous coronary intervention within the last 12 weeks, myocardial infarction (STEMI and non-STEMI), unstable angina or any kind of stroke within the last 12 weeks, acute myocarditis within the last 12 weeks, pregnancy or breast feeding

A total of 941 HFrEF patients were screened (81.6% male, mean age 65.5 ± 12 years); 106 were referred for PSG of whom 30 did not have moderate-to-severe OSA, leaving 76 patients who were enrolled in the study and randomized to the APAP or control group (Fig. 2). The per-protocol final analysis included 58 patients who completed the study (Fig. 2). The most common reason for exclusion was withdrew of consent, protocol deviation by the patient and inability to perform appropriate cardiopulmonary exercise testing (Fig. 2).

Table 1 provides a summary of demographic and clinical characteristics of the per-protocol population. The study groups were well balanced, with no statistically significant differences between patients randomized to the APAP (*n* = 25) or control (*n* = 33) groups (Table 1).

**Table 1:** Baseline demographics and clinical characteristics

	Overall	Control	APAP	*p* value
	(*n* = 58)	(*n* = 33)	(*n* = 25)	
Age, years	66.1 ± 10.0	64.9 ± 10.1	67.4 ± 9.8	0.276
Male	62 (81.6)	32 (82.1)	30 (81.1)	0.913
Height, m	1.74 ± 0.09	1.74 ± 0.09	1.74 ± 0.09	0.874
Weight, kg	92.4 ± 20.5	93.8 ± 21.4	90.9 ± 20.0	0.553
BMI, kg/m^2^	29.1 ± 5.7	30.7 ± 5.9	30.0 ± 5.5	0.576
NYHA class				
II	46 (60.5)	23 (59.0)	23 (62.2)	0.804
III	4 (5.3)	2 (5.1)	2 (5.4)	
IV	1 (1.3)	1 (2.6)	0 (0)	
Nocturia, episodes/night		1.68 ± 1.33	1.73 ± 1.67	0.896
Heart rate, beats/min		67.0 ± 12.6	70.8 ± 9.0	0.897
Systolic BP, mmHg		127.5 ± 21.8	130.3 ± 19.5	0.553
Diastolic BP, mmHg		72.5 ± 11.1	69.6 ± 13.5	0.306
Atrial fibrillation	9 (11.8)	3 (7.7)	6 (16.2)	0.516
Ischemic etiology	39 (51.3)	23 (59.0)	16 (43.2)	0.170
Implanted device				
Pacemaker	4 (5.3)	2 (5.1)	2 (5.4)	0.851
ICD	13 (17.1)	8 (20.5)	5 (13.5)	
CRT(P/D)	19 (25.0)	10 (25.6)	9 (24.3)	
Medication				
ACEI, ARB	64 (84.2)	32 (82.1)	32 (86.5)	0.596
Βeta blocker	71 (93.4)	39 (100)	32 (86.5)	0.018
Diuretics	53 (69.7)	29 (74.4)	24 (64.9)	0.368
Spironolactone or eplerenone	46 (60.5)	25 (64.1)	21 (56.8)	0.513
Cardiac glycosides	4 (5.3)	1 (2.6)	3 (8.1)	0.279
Amiodarone	15 (19.7)	7 (17.9)	8 (21.6)	0.688

Values are mean ± SD, or *n* (%)

*ACEI* angiotensin-converting enzyme inhibitor, *ARB* angiotensin II receptor blocker, *BMI* body mass, *BP* blood pressure, *CRT(D)* cardiac resynchronization therapy (defibrillator), *ICD* implantable cardioverter-defibrillator, *NYHA* New York Heart Association

### Primary endpoint

Out of the three predefined primary endpoints, peak VO_2_ and percent-predicted peak VO_2_ improved significantly from baseline to 6 months in the APAP group, but not in the control group (Table 2). The change in peak VO_2_ from baseline to 6 months was 1.15 mL/kg/min higher in the APAP group compared with control, but the between-group difference did not achieve statistical significance (*p* = 0.064) (Table 2). This equated to an average improvement of 0.24 mL/kg/min per week (95% confidence interval 0.03–0.45; *p* = 0.03) over the 6-month follow-up in the APAP therapy versus control group, but comparison was not statistically significant in a secondary analysis including three patients that had 3-month, but not 6-month, data available (change in peak VO_2_ of 0.21 mL/kg/min per week; *p* = 0.053). In contrast, improvement in the predefined primary endpoint percent-predicted peak VO_2_ was significantly different between the APAP and control group (6.58%; *p* = 0.01) (Table 2).

**Table 2:** Cardiopulmonary exercise capacity (per-protocol analysis)

	Baseline	6 months	*p* value	Change from baseline	Between-group difference for change from baseline (*p* value)
Primary endpoints					
Percent-predicted VO_2_ peak, %					
Control (*n* = 33)	69.6 ± 20.4	68.6 ± 20.0	0.576	– 1.03 ± 10.5	0.010
APAP (*n* = 25)	67.4 ± 17.0	73.2 ± 19.2	0.003	+ 5.76 ± 8.8	
VO_2_-AT, mL/kg/min					
Control (*n* = 33)	12.99 ± 2.9	12.92 ± 2.9	0.863	– 0.06 ± 2.12	0.088
APAP (*n* = 25)	12.72 ± 3.4	13.67 ± 3.3	0.049	+ 0.95 ± 2.30	
Peak VO_2_, mL/kg/min					
Control (*n* = 33)	15.71 ± 3.9	15.68 ± 4.3	0.956	– 0.02 ± 2.48	0.064
APAP (*n* = 25)	15.01 ± 3.8	16.17 ± 4.6	0.014	+ 1.16 ± 2.18	
Secondary endpoints					
VE/VCO_2_					
Control (*n* = 33)	29.9 ± 2.8	29.5 ± 3.1	0.457	– 0.40 ± 0.52	0.012
APAP (*n* = 25)	30.8 ± 6.4	30.3 ± 6.8	0.410	– 4.3 ± 2.17	
Workload, W					
Control (*n* = 33)	103.0 ± 34.4	100.5 ± 33.6	0.434	– 2.52 ± 18.2	0.285
APAP (*n* = 25)	103.4 ± 35.7	107.3 ± 43.8	0.478	+ 3.88 ± 26.9	

Values are mean ± SD

*APAP* automatic positive airway pressure, *Peak VO*_*2*_ peak oxygen consumption, *VE/VCO*_*2*_ measure of ventilatory efficiency (minute ventilation relative to carbon dioxide exhalation), *VO*_*2*_*-AT* maximal oxygen consumption at the anaerobic threshold

### Secondary endpoints

The MLHFQ score and LVEF improved significantly from baseline to 6 months in the APAP group but not the control group, whereas the ESS score and 6MWD did not change significantly from baseline in either treatment group (Table 3).

**Table 3:** Functional capacity, sleepiness and quality of life (per-protocol analysis)

	Baseline	6 months	*p* value	Change from baseline	Between-group difference for change from baseline for APAP vs control (*p* value)
6MWD, m					
Control (*n* = 33)	409.6 ± 104.7	419.6 ± 100.4	0.566	9.3 ± 99.4	0.631
APAP (*n* = 25)	406.2 ± 66.4	390.8 ± 85.0	0.170	– 15.4 ± 72.3	
MLHFQ score					
Control (*n* = 33)	30.88 ± 18.79	29.38 ± 19.36	0.550	– 1.3 ± 18.6	0.478
APAP (*n* = 25)	29.37 ± 20.14	24.00 ± 19.66	0.037	– 5.1 ± 19.4	
ESS score					
Control (*n* = 33)	7.24 ± 3.91	7.09 ± 5.32	0.830	– 0.1 ± 4.1	0.841
APAP (*n* = 25)	7.04 ± 4.15	6.78 ± 3.40	0.743	– 0.2 ± 3.6	
LVEF, %					
Control (*n* = 33)	36.31 ± 6.91	39.23 ± 9.41	0.086	2.9 ± 7.8	0.132
APAP (*n* = 25)	39.29 ± 6.51	44.35 ± 8.96	0.001	4.1 ± 7.7	

Values are mean ± SD

*6MWT* six-minute walk test, *APAP* automatic positive airway pressure, *ESS* Epworth sleepiness scale, *LVEF* left ventricular ejection fraction, *MLHFQ* Minnesota Living with Heart Failure Questionnaire

On echocardiography, changes from baseline in right ventricular global longitudinal strain were significantly greater in the APAP group (− 16.8 ± 7.4% to − 20.1 ± 6.1%) compared with the control group (− 19.0 ± 3.6% to − 18.6 ± 3.6%; *p* = 0.04), as were changes in left ventricular global longitudinal strain with APAP (− 9.0 ± 2.8% to − 10.6 ± 2.8%) versus control (− 8.8 ± 2.8% to − 9.3 ± 2.8%; *p* < 0.001). In addition, LVEF improved to a significantly greater extent in the APAP group (from 38 ± 9% at baseline to 40 ± 9% at 6 months) compared with controls (40 ± 9% to 40 ± 8%; *p* < 0.01). No statistically significant changes in the functional area of change, tricuspid annular plane systolic excursion, left atrial volume index or right atrial volume index were documented during the study.

The AHI decreased significantly from baseline to 6 months in the APAP group (from 34 ± 17/h to 9 ± 8/h; *p* < 0.001), but remained unchanged in the control group (from 35 ± 13/h to 33 ± 20/h). Sleep stages were not different between groups: REM phases (18.3 ± 7.9% of total sleep time), N1 (26.8 ± 15.1% of total sleep time), N2 (47.8 ± 13.9% of total sleep time) and N3 (4.7 ± 6.1% of total sleep time). Portion of central respiratory events and Cheyne–Stokes respiration has been found low, as required by inclusion criteria. Average APAP pressure applied was 15.6 ± 2.2 mmHg, the maximum pressure sealed at 20 mmHg. Our study protocol implied close patient follow-up, resulting in momentous compliance and an APAP device use of an average of 6 ± 1.6 h per night. Days of APAP use above 4 h per night was 61 ± 28%. Minimum oxygen saturation increased slightly in the control group but improved to a significantly greater extent in the APAP group (Table 4). Average oxygen saturation, mean oxygen desaturation, maximum apnea duration and maximum hypopnea duration all significantly improved from baseline and compared with the control group during APAP therapy (Table 4). Lung function remained stable throughout the study in both groups.

**Table 4:** Respiratory parameters (per-protocol analysis)

	Baseline	6 months	p value within group	Change from baseline	Between-group difference for change from baseline for APAP vs control (*p* value)
Average oxygen saturation, %					
Control (*n* = 33)	92.03 ± 2.23	92.00 ± 2.97	0.857	− 0.06 ± 1.92	0.006
APAP (*n* = 25)	92.47 ± 2.62	93.82 ± 1.92	0.001	+ 1.34 ± 1.85	
Minimum oxygen saturation, %					
Control (*n* = 33)	78.41 ± 5.56	79.84 ± 6.12	0.038	+ 1.44 ± 3.76	< 0.001
APAP (*n* = 25)	78.52 ± 6.65	87.52 ± 3.40	< 0.001	+ 9.00 ± 6.11	
Mean oxygen desaturation, %					
Control (*n* = 33)	5.77 ± 1.89	5.78 ± 2.37	0.957	+ 0.02 ± 1.62	< 0.001
APAP (*n* = 25)	5.75 ± 1.74	3.92 ± 0.55	< 0.001	− 1.83 ± 1.82	
Maximum apnea duration, s					
Control (*n* = 33)	45.91 ± 22.66	42.15 ± 22.85	0.428	− 3.76 ± 26.9	< 0.001
APAP (*n* = 25)	51.66 ± 27.22	20.86 ± 12.47	< 0.001	− 30.80 ± 29.20	
Maximum hypopnea duration, s					
Control (*n* = 33)	73.85 ± 40.43	66.58 ± 27.08	0.285	− 7.27 ± 38.50	< 0.001
APAP (*n* = 25)	81.05 ± 27.84	46.52 ± 22.02	< 0.001	− 34.53 ± 29.90	

Values are mean ± SD

*APAP* automatic positive airway pressure

## Discussion

This is the first randomized controlled trial to show improvement in percent-predicted peak VO_2_ as a primary study endpoint in APAP therapy in HFrEF patients with OSA determined in cardiopulmonary exercise capacity testing (Fig. 1). Moreover, this study shows enhanced global cardiac function after 6 months of APAP therapy and our study demonstrates statistically significant and clinically meaningful improvements of APAP therapy for polysomnography parameters, functional variables, along with parallel improvements in echocardiographic parameters of both right and left ventricular function (Tables 2, 3 and 4).

A previous randomized trial evaluated the effects of 6 weeks of APAP in 26 patients with chronic stable HFrEF but failed to document any significant improvement in cardiopulmonary exercise capacity and quality of life, and did not report respiratory or sleep parameters [21]. However, the treatment duration in that study (6 weeks) was much shorter than in our study and may have been insufficient to show any benefit of APAP on cardiopulmonary exercise. Furthermore, device use in the previous study was low, averaging 3.5 h per night and this might also have been not enough to obtain potential benefit from APAP therapy, while patients in our study revealed good device use averaging 6.0 ± 1.6 h per night. The importance of device use in realizing the beneficial effects of APAP was indicated in our study by the fact that the per-protocol analysis showed a significant improvement in percent-predicted peak VO_2_ in the APAP group, but a secondary analysis including patients with exercise capacity data at only 3 months failed to reach statistical significance. This suggests that only patients with ongoing use of APAP obtained greater benefit from therapy.

Our results were similar to those from another randomized study investigating use of CPAP for 3 months in patients with HF and LVEF < 55% [22]. As in our trial of APAP, CPAP treatment was associated with significant improvements in LVEF and quality of life compared with controls, and the magnitude of changes in LVEF was similar to that seen in our study (≈ 5%) [22]. Improvements of > 5% in the LVEF during drug therapy for HF have been shown to predict improved survival and decreased HF-related hospitalization [23]. Therefore, the effects of APAP on left ventricular function documented in our study have the potential to be clinically meaningful.

Another important finding from this study is that patients treated with APAP had a significant improvement in average oxygen saturation compared with baseline and the control group. This is also clinically relevant, because hypoxemic burden has been shown to be a robust and independent predictor of mortality in HFrEF [11].

Multiple comorbidities are common in patients with HFrEF and these show a complex and bidirectional relationship with HF [24]. Management of these comorbidities, including SDB, is a key component of modern holistic care for HFrEF [25]. OSA is often undiagnosed in patients with HFrEF but has been shown to be associated with increased mortality [26]. It can be difficult to identify typical OSA symptoms in HFrEF patients because the two conditions share a number of common symptoms, including fatigue, sleepiness and exercise intolerance. This means that traditional tools, such as the ESS, often fail to identify OSA in patients with cardiovascular disease [27]. In our study, APAP had no effect on the ESS score, but patients did not have excessive daytime sleepiness at baseline (mean ESS score of just over 7 whereas scores > 10 indicate sleepiness).

The results of cardiopulmonary exercise testing have been shown to predict prognosis in patients with HFrEF [28]. In particular, peak VO_2_ is an important predictor of survival in HF [29]. In the multicenter Heart Failure and a Controlled Trial Investigating Outcomes of Exercise Training (HF-ACTION) trial [30], exercise training was associated with a 6% increase in peak VO_2_ over 3 months and a significantly lower risk of cardiovascular mortality or HF hospitalization after adjustment for highly prognostic baseline characteristics (hazard ratio versus control of 0.85, 95% confidence interval 0.74–0.99; *p* = 0.03) [30]. Our study patients had an initial peak VO_2_ of 15.01 mL/kg/min and through APAP intervention peak VO_2_ improved to 16.17 mL/kg/min, which represents a total improvement of 1.16 ± 2.18 mL/kg/min (7.7%). Although we missed the aspired 10% difference in peak VO_2_ values between APAP and control group, we can demonstrate a clinically important 7.7% improvement in our trial, because each 6% increase in peak VO_2_ over only 3 months was shown to be accompanied with significant reduction in the risk of all-cause mortality and all-cause hospitalization [31]. Another study showed that peak VO_2_ was a strong predictor of mortality [31]. After adjustment for other significant predictors, each 6% increase in peak VO_2_ was associated with a 5% reduction in the risk of all-cause mortality and all-cause hospitalization (*p* < 0.001) [31].

Although our study failed to demonstrate statistical significance for all three pre-defined primary endpoints [13], our study results picture a very important finding with significant improvement specifically for percent-predicted peak VO_2_. Percent-predicted peak VO_2_ has been shown to be superior to other endpoints. Ross Arena et al clearly demonstrate percent-predicted peak VO_2_ to be the most interesting variable [32] because in their analysis, percent-predicted peak VO_2_ value derived from the Wasserman/ Hansen equations had outperformed all other expressions of cardiopulmonary exercise testing variables [32]. This is of particular interest because our study population reveals small differences in age and weight. Although these baseline characteristic differences are not statistically significantly different, one may argue that they may influence our study results. Percent-predicted peak VO_2_ controls for such variables, making percent-predicted peak VO_2_ the most important variable, supporting the meaningful impact of our study result as this endpoint has effect on mortality [32].

The largest trial on mortality in this field is the SERVE HF [33] study which investigated predominant central sleep apnea in HFrEF patients through application of adaptive servo-ventilation. Our trial studied predominant obstructive sleep apnea in HFrEF through application of APAP therapy, which makes comparison of both studies extremely difficult as two different disease patterns have been explored. However, the SERVE HF study [33] revealed adverse events for the use of adaptive servo-ventilation, while our study results are encouraging APAP treatment in HFrEF patients with predominant obstructive sleep apnea and our results yield hope for further trials to improve outcome in HFrEF patients by treating obstructive sleep apnea.

Our randomized, controlled clinical pilot study showed that use of APAP therapy for 6 months in patients with HFrEF and OSA had beneficial effects, not only on an important primary endpoint, but also on a number of surrogate markers for cardiovascular outcome. Nevertheless, there is a need for more randomized controlled trials to confirm the effects of positive airway pressure therapy on cardiopulmonary exercise capacity, quality of life, global cardiac function, and hypoxemic burden, and to determine whether these improvements translate into better outcomes for HFrEF patients with OSA.

## Conclusion

APAP intervention was shown to significantly improve outcome compared to control group, represented in percent-predicted peak VO_2_, an established surrogate marker for cardiovascular prognosis in HFrEF. APAP has additional beneficial effects on hypoxemia, cardiac function and quality of life.

## Limitations

Although this is a randomized controlled trial, it is conducted as a single-center trial and its results may not be transmissible universally. This study had a follow-up period of only 6 months, and that is why no information is available on additional heart failure outcome through APAP therapy. Our good APAP therapy group compliance, presumably accountable for the close mentoring of our sleep lab patients, may not be realizable and applicable in every outpatient cohort and less therapy compliance may diminish our findings. Nevertheless, we unexpectedly missed 6 patients in the control and 12 patients in the APAP therapy group for different reasons (Fig. 2). Unfortunately, study funding did not allow replenishing these dropouts, making additional patient inclusions hereinafter impossible, and that is why our conceived power calculation has not been met. Dropouts and premature discontinuation for limited funding of our trial may be accountable reasons why all three predefined primary study endpoints could not be met. More data, long-term and larger studies are needed to determine the impact of our findings on heart failure outcome, including mortality.

## References

[1] Fox H, Purucker HC, Holzhacker I, Tebtmann U, Bitter T, Horstkotte D, Graml A, Woehrle H, Oldenburg O (2016). Prevalence of sleep-disordered breathing and patient characteristics in a coronary artery disease cohort undergoing cardiovascular rehabilitation. J Cardiopulm Rehabil Prev.

[2] Oldenburg O, Lamp B, Faber L, Teschler H, Horstkotte D, Topfer V (2007). Sleep-disordered breathing in patients with symptomatic heart failure: a contemporary study of prevalence in and characteristics of 700 patients. Eur J Heart Fail.

[3] Omran H, Bitter T, Horstkotte D, Oldenburg O, Fox H (2018). Characteristics and circadian distribution of cardiac arrhythmias in patients with heart failure and sleep-disordered breathing. Clin Res Cardiol.

[4] Shahar E, Whitney CW, Redline S, Lee ET, Newman AB, Nieto FJ, O’Connor GT, Boland LL, Schwartz JE, Samet JM (2001). Sleep-disordered breathing and cardiovascular disease: cross-sectional results of the Sleep Heart Health Study. Am J Respir Crit Care Med.

[5] Khayat R, Jarjoura D, Porter K, Sow A, Wannemacher J, Dohar R, Pleister A, Abraham WT (2015). Sleep disordered breathing and post-discharge mortality in patients with acute heart failure. Eur Heart J.

[6] Fox H, Bitter T, Horstkotte D, Oldenburg O (2016). Cardioversion of atrial fibrillation or atrial flutter into sinus rhythm reduces nocturnal central respiratory events and unmasks obstructive sleep apnoea. Clin Res Cardiol.

[7] Mayer G, Arzt M, Braumann B, Ficker JH, Fietze I, Frohnhofen H, Galetke W, Maurer JT, Orth M, Penzel T, Pistner H, Randerath W, Rosslein M, Sitter H, Stuck BA (2017). German S3 guideline nonrestorative sleep/sleep disorders, chapter “Sleep-Related Breathing Disorders in Adults,” short version: German Sleep Society (Deutsche Gesellschaft fur Schlafforschung und Schlafmedizin, DGSM). Somnologie (Berl).

[8] Fox H, Bitter T, Horstkotte D, Oldenburg O (2017). Sleep-disordered breathing and arrhythmia in heart failure patients. Sleep Med Clin.

[9] Spaak J, Egri ZJ, Kubo T, Yu E, Ando S, Kaneko Y, Usui K, Bradley TD, Floras JS (2005). Muscle sympathetic nerve activity during wakefulness in heart failure patients with and without sleep apnea. Hypertension.

[10] Liu F, Chan AQ, Wang B (2016). Obstructive sleep apnea-hypopnea and cardiovascular diseases in adults. Cardiol Plus.

[11] Oldenburg O, Wellmann B, Buchholz A, Bitter T, Fox H, Thiem U, Horstkotte D, Wegscheider K (2016). Nocturnal hypoxaemia is associated with increased mortality in stable heart failure patients. Eur Heart J.

[12] Sullivan CE, Issa FG, Berthon-Jones M, Eves L (1981). Reversal of obstructive sleep apnoea by continuous positive airway pressure applied through the nares. Lancet.

[13] Oldenburg O, Fox H, Wellmann B, Thiem U, Horstkotte D, Bitter T (2017). Automatic positive airway pressure for treatment of obstructive sleep apnea in heart failure. Somnologie.

[14] Sharma S, Fox H, Aguilar F, Mukhtar U, Willes L, Bozorgnia B, Bitter T, Oldenburg O (2019). Auto positive airway pressure therapy reduces pulmonary pressures in adults admitted for acute heart failure with pulmonary hypertension and obstructive sleep apnea. The ASAP-HF pilot trial. Sleep.

[15] Spiesshofer J, Fox H, Lehmann R, Efken C, Heinrich J, Bitter T, Korber B, Horstkotte D, Oldenburg O (2016). Heterogenous haemodynamic effects of adaptive servoventilation therapy in sleeping patients with heart failure and Cheyne-Stokes respiration compared to healthy volunteers. Heart Vessels.

[16] McEvoy RD, Antic NA, Heeley E, Luo Y, Ou Q, Zhang X, Mediano O, Chen R, Drager LF, Liu Z, Chen G, Du B, McArdle N, Mukherjee S, Tripathi M, Billot L, Li Q, Lorenzi-Filho G, Barbe F, Redline S, Wang J, Arima H, Neal B, White DP, Grunstein RR, Zhong N, Anderson CS, S Investigators, Coordinators (2016). CPAP for prevention of cardiovascular events in obstructive sleep apnea. N Engl J Med.

[17] Becker HF, Jerrentrup A, Ploch T, Grote L, Penzel T, Sullivan CE, Peter JH (2003). Effect of nasal continuous positive airway pressure treatment on blood pressure in patients with obstructive sleep apnea. Circulation.

[18] Kanagala R, Murali NS, Friedman PA, Ammash NM, Gersh BJ, Ballman KV, Shamsuzzaman AS, Somers VK (2003). Obstructive sleep apnea and the recurrence of atrial fibrillation. Circulation.

[19] Corra U, Piepoli MF, Adamopoulos S, Agostoni P, Coats AJ, Conraads V, Lambrinou E, Pieske B, Piotrowicz E, Schmid JP, Seferovic PM, Anker SD, Filippatos G, Ponikowski PP (2014). Cardiopulmonary exercise testing in systolic heart failure in 2014: the evolving prognostic role: a position paper from the committee on exercise physiology and training of the heart failure association of the ESC. Eur J Heart Fail.

[20] Yagihara F, Lorenzi-Filho G, Santos-Silva R (2017). Nasal dilator strip is an effective placebo intervention for severe obstructive sleep apnea. J Clin Sleep Med.

[21] Smith LA, Vennelle M, Gardner RS, McDonagh TA, Denvir MA, Douglas NJ, Newby DE (2007). Auto-titrating continuous positive airway pressure therapy in patients with chronic heart failure and obstructive sleep apnoea: a randomized placebo-controlled trial. Eur Heart J.

[22] Mansfield DR, Gollogly NC, Kaye DM, Richardson M, Bergin P, Naughton MT (2004). Controlled trial of continuous positive airway pressure in obstructive sleep apnea and heart failure. Am J Respir Crit Care Med.

[23] Breathett K, Allen LA, Udelson J, Davis G, Bristow M (2016). Changes in left ventricular ejection fraction predict survival and hospitalization in heart failure with reduced ejection fraction. Circ Heart Fail.

[24] Ather S, Chan W, Bozkurt B, Aguilar D, Ramasubbu K, Zachariah AA, Wehrens XH, Deswal A (2012). Impact of noncardiac comorbidities on morbidity and mortality in a predominantly male population with heart failure and preserved versus reduced ejection fraction. J Am Coll Cardiol.

[25] Feldmann C, Ertl G, Angermann CE (2014). Holistic therapy of chronic heart failure. Internist (Berl).

[26] Wang H, Parker JD, Newton GE, Floras JS, Mak S, Chiu KL, Ruttanaumpawan P, Tomlinson G, Bradley TD (2007). Influence of obstructive sleep apnea on mortality in patients with heart failure. J Am Coll Cardiol.

[27] Reuter H, Herkenrath S, Treml M, Halbach M, Steven D, Frank K, Castrogiovanni A, Kietzmann I, Baldus S, Randerath WJ (2018). Sleep-disordered breathing in patients with cardiovascular diseases cannot be detected by ESS, STOP-BANG, and Berlin questionnaires. Clin Res Cardiol.

[28] Cahalin LP, Chase P, Arena R, Myers J, Bensimhon D, Peberdy MA, Ashley E, West E, Forman DE, Pinkstaff S, Lavie CJ, Guazzi M (2013). A meta-analysis of the prognostic significance of cardiopulmonary exercise testing in patients with heart failure. Heart Fail Rev.

[29] Myers J, Gullestad L, Vagelos R, Do D, Bellin D, Ross H, Fowler MB (2000). Cardiopulmonary exercise testing and prognosis in severe heart failure: 14 mL/kg/min revisited. Am Heart J.

[30] O’Connor CM, Whellan DJ, Lee KL, Keteyian SJ, Cooper LS, Ellis SJ, Leifer ES, Kraus WE, Kitzman DW, Blumenthal JA, Rendall DS, Miller NH, Fleg JL, Schulman KA, McKelvie RS, Zannad F, Pina IL, Investigators H-A (2009). Efficacy and safety of exercise training in patients with chronic heart failure: HF-ACTION randomized controlled trial. JAMA.

[31] Swank AM, Horton J, Fleg JL, Fonarow GC, Keteyian S, Goldberg L, Wolfel G, Handberg EM, Bensimhon D, Illiou MC, Vest M, Ewald G, Blackburn G, Leifer E, Cooper L, Kraus WE, Investigators H-A (2012). Modest increase in peak VO_2_ is related to better clinical outcomes in chronic heart failure patients: results from heart failure and a controlled trial to investigate outcomes of exercise training. Circ Heart Fail.

[32] Arena R, Myers J, Abella J, Pinkstaff S, Brubaker P, Moore B, Kitzman D, Peberdy MA, Bensimhon D, Chase P, Forman D, West E, Guazzi M (2009). Determining the preferred percent-predicted equation for peak oxygen consumption in patients with heart failure. Circ Heart Fail.

[33] Cowie MR, Woehrle H, Wegscheider K, Angermann C, d’Ortho MP, Erdmann E, Levy P, Simonds AK, Somers VK, Zannad F, Teschler H (2015). Adaptive servo-ventilation for central sleep apnea in systolic heart failure. N Engl J Med.

